# A South African Investigation Into Etiological and Background Factors in Developmental Dyslexia

**DOI:** 10.1002/dys.70039

**Published:** 2026-07-14

**Authors:** Salomé Geertsema, Eileen Olivier, Cassidy Shaw, Isabel Turner, Dandelion Valkenburg, Kallyn van der Spuy, Sandra Stark, Mia Le Roux, Marien Alet Graham, Heidi Mapisa

**Affiliations:** ^1^ Department of Speech‐Language Pathology and Audiology University of Pretoria Pretoria South Africa; ^2^ Department of Mathematics Education, College of Education University of South Africa Pretoria South Africa; ^3^ Department of Speech‐Language Pathology and Audiology Sefako Makgatho Health Sciences University Ga‐Rankuwa South Africa

**Keywords:** background factors, developmental dyslexia, diagnosis, etiological factors, South Africa

## Abstract

Understanding the etiological and background factors associated with developmental dyslexia (DD) is crucial for effective early identification, perceptive support, informed therapist management, and differentiated teacher instruction. A retrospective, cross‐sectional, and comparative research design was employed to investigate these factors in the South African population with DD using existing data from a professional academy for the assessment and diagnosis of DD. Four thousand three hundred and seventy school‐aged individuals were included. Data were collected across South Africa through the academy's standardised and validated diagnostic tool. Caregiver consent and child assent for the anonymised use of data for further research were obtained before collection. Specific factors of late speech development, prior speech therapy, audiological consultations, negative emotions (anger, frustration, and diagnosed depression), and gender were investigated. Statistical analyses revealed specific significant associations between these factors and different subtypes of DD. Findings underscore the importance of noting the various etiological and background factors associated with DD subtypes, which can inform knowledgeable detection and sympathetic support in classroom settings and subsequent referral and intervention strategies.

## Practitioner Points

- Understanding the etiological and background factors associated with developmental dyslexia (DD) is crucial for effective early identification, perceptive support, informed therapist management, and differentiated teacher instruction.
- Factors of late speech development, prior speech therapy, audiological consultations, anger, frustration, depression, and gender were correlated in this study.
- Findings underscore the importance of role players taking note of various etiological and background factors associated with DD subtypes.
- These factors will inform educated early detection and subsequent additional referral and intervention strategies.
- Educators and professionals may also develop more sympathetic support in classroom settings, and not only attribute symptoms to poor behaviour.

## 1 Introduction

Specific learning disorder (SLD) is a neurodevelopmental condition characterised by persistent difficulties in acquiring and using academic skills despite adequate instruction and average or above‐average intellectual ability (Geertsema et al. 2022). Children with SLDs, such as developmental dyslexia (DD), present with learning difficulties and impaired academic skills, manifesting in one or more symptoms: arduous word reading, difficulty with reading comprehension, spelling, written expression, mastering number sense, number facts, and analytical reasoning (Grigorenko et al. 2020). The disorder's physical impact is evident in classroom participation, everyday activities, and social interactions (Geertsema et al. 2022). The academic skills of these children with dyslexia (CWD) are quantifiably below those expected for their chronological age group. This can cause significant limitations in educational and occupational functioning.

In the Diagnostic and Statistical Manual of Mental Disorders, Fifth Edition, Text Revision (DSM‐5‐TR) (American Psychiatric Association 2022), DD is classified as an SLD with reading difficulties and is defined by difficulties in accurate word reading, reading fluency, and spelling. The DSM‐5‐TR specifies diagnostic criteria and severity levels (mild, moderate, and severe) but does not endorse etiological or cognitive categorisations. Consequently, DSM‐5‐TR functions as a diagnostic system rather than a clarification of underlying processes. Importantly, however, DD is widely recognised as a varied condition (Grigorenko et al. 2020; Stark et al. 2022; Compton 2021), and investigations into these underlying and contributing factors are therefore essential.

CWD may exhibit diverging patterns of strengths and weaknesses across phonological, orthographic, motor/graphomotor, attentional, and sensory domains (Grigorenko et al. 2020). This diversity has resulted in different explanatory models, including phonological deficit theories, multiple‐deficit frameworks, and neurobiological explanations that emphasise variations in auditory, visual, or sensorimotor processing (Stark et al. 2022; Compton 2021). Contemporary consensus increasingly supports a multiple‐deficit viewpoint, in which DD arises from the interaction of multiple risk factors rather than a single fundamental impairment (Compton 2021).

DD frequently occurs alongside several neurodevelopmental disorders, including attention‐deficit/hyperactivity disorder (ADHD), developmental coordination disorder, autism spectrum disorder (ASD), and unusual sensory processing profiles (Brimo et al. 2021). Emotional and behavioural problems—such as anxiety, frustration, low self‐worth, and signs of depression—are commonly recognised and often viewed as consequences of persistent academic difficulties and constant feelings of failure (Huang et al. 2020; Livingston et al. 2018). Factors such as socioeconomic background, teaching quality, home literacy environment, and friendships shape the experiences of those with DD. Specifically, environmental factors may affect them even more than they affect their neurotypical peers. These include culture, social strata, schooling characteristics, family literacy environments, and peer influences (Krishnan et al. 2016). Hence, a detailed assessment must be conducted in CWD to determine the presence, severity, subtypes, and related factors. Such evaluations usually include clinical considerations and psychometric assessments. Additionally, these measurements should include a direct assessment of the ability to decode, encode, and write (Zoubrinetzky et al. 2014) while adhering to DSM‐5‐TR criteria for SLD (Stark et al. 2022). Consequently, these related factors underscore the importance of a thorough biopsychosocial assessment.

In this context, the present study distinguishes between DSM‐5‐TR diagnosis and descriptive functional evaluation. Every participant in this research met the DSM‐5‐TR criteria for SLD, characterised by reading challenges. The DD “subtypes” referenced in this study—namely dysnemkinesia, dysphonesia, dyseidesia, and their combinations—are not recognised as diagnostic categories and do not change or supplant DSM‐5‐TR criteria. Instead, they act as descriptive functional profiles intended to depict the main processing traits observed in a pre‐established DSM‐5‐TR diagnosis (Stark et al. 2022). In theory, these descriptive profiles correspond to categories identified in worldwide DD studies: dysphonesia signifies difficulties in phonological processing and phoneme–grapheme integration; dyseidesia reflects reduced proficiency in visual–orthographic processing and whole‐word recognition; whereas dysnemkinesia represents challenges in motor–procedural learning related to the creation and automation of written symbols. As intended in this manuscript, the profiling system represents a clinical application of recognised cognitive areas employed to inform intervention techniques and modifications, without implying separate categories (Stark et al. 2022).

Recent international research increasingly conceptualises DD as a multifactorial neurodevelopmental condition arising from the interaction of genetic susceptibility, atypical neural connectivity, phonological processing inefficiencies, and domain‐general cognitive mechanisms such as processing speed and executive functioning (Shaywitz et al. 2021; Kim 2021). Advances in neuroimaging continue to demonstrate altered activation patterns within left‐hemispheric temporo‐parietal and occipito‐temporal reading networks, alongside reduced white‐matter integrity in tracts supporting phonological decoding and orthographic mapping (Liapi et al. 2024). Contemporary models therefore emphasise that dyslexia is not attributable to a single deficit but reflects distributed network differences affecting auditory‐temporal processing, visual‐orthographic integration, and motor‐graphomotor encoding systems. These findings reinforce the importance of examining etiological indicators that align with specific cognitive‐linguistic domains underlying distinct DD subtypes.

Parallel to advances in causal research, recent large‐scale reviews have confirmed that CWD experience significantly elevated rates of anxiety, depression, school‐related distress, and emotional dysregulation compared to typically developing peers (Wilmot et al. 2023). Emerging longitudinal evidence further suggests that internalising symptoms may intensify over time when reading difficulties remain unidentified or unsupported, particularly in learners with more complex or combined deficit profiles (Granocchio et al. 2023). Emotional comorbidities are therefore increasingly viewed not merely as secondary reactions but as interacting developmental consequences that can exacerbate academic impairment and functional outcomes. Within this evolving international framework, investigating emotional variables alongside neurodevelopmental indicators is essential for a comprehensive understanding of DD subtypes and their real‐world impact.

In South Africa, practitioners registered with the Health Professions Council conduct DD evaluations using standardised and validated instruments. Assessment techniques include caregiver reports, direct evaluation of decoding, encoding, and writing skills, and adherence to DSM‐5‐TR criteria for SLD diagnoses involving reading challenges. In this study, functional profiles were developed only after meeting diagnostic criteria and were used to explore associations with selected background factors rather than to make causal interpretations. The DSM‐5‐TR explicitly allows for the inclusion of clinical specifiers, profiles, and descriptive qualifiers alongside diagnostic coding, provided they do not contradict the diagnostic criteria (APA 2022). In line with a multiple‐deficit and biopsychosocial perspective, this research examined various background and related elements that could cluster with particular functional profiles.

A focus of interest was the history of hearing‐related problems. Research indicates that some CWD struggle to perceive spoken language in noisy environments, emphasising differences in central auditory and phonological processing rather than issues with peripheral hearing (Werfel et al. 2020). Werfel and colleagues found that 71% of their experimental group failed the hearing screening and exhibited decoding deficits. These challenges relate to reduced temporal auditory processing and poorer phonological representations, which may affect phoneme discrimination and decoding (Rahimi et al. 2022; Habib 2021). The reasons for including the primary type of dysphonesia are therefore ostensible. Additionally, the possibility of parents seeking an audiologist to address the difficulties their children may experience seems a likely starting point.

Likewise, slow or unusual speech and language development has been identified as a sign of potential future literacy difficulties. Late talkers often exhibit weaknesses in phonology, grammar, and vocabulary, and, subsequently, in reading accuracy and fluency (Dynak et al. 2021). The parallels between initial language delay and later DD warrant viewing speech development history as a significant related element. Parents might notice that their child is not meeting developmental milestones for speech and language, such as babbling, saying first words, or combining words into simple sentences at the expected ages. They, and later the educators, may also observe difficulties with pronunciation, limited vocabulary, or problems understanding and following directions (Bernthal et al. 2018). A deferred speech‐language therapist (SLT) referral to emergent literacy and literacy skills usually lies with the informed educator or parent (Bridges and Kelley 2023). However, referrals may be compromised by anecdotal and scientific evidence indicating that several SLTs in South Africa (Erasmus et al. 2013; Geertsema and Le Roux 2020) and abroad (Bridges and Kelley 2023) lack confidence in working with children who have general or specific literacy difficulties. Even more so, many of our local clinicians report not assessing or treating CWD, and educators are unsure what to look for when considering possible referral factors (Erasmus et al. 2013; Geertsema and Le Roux 2020). Thus, the exploration of prior SLT services was conducted as an additional contextual element that could suggest earlier identification.

The emotional impact of DD is also considered. Persistent academic difficulties place CWD at an increased likelihood of negative emotional experiences, including anger, frustration, anxiety, and depressive symptoms (Livingston et al. 2018). Although these emotional responses are not intrinsic diagnostic characteristics of DD, they are frequently discussed in relation to motivation, engagement in class, and general well‐being; understanding how emotional factors relate to functional profiles could help educators and clinicians provide more effective support. DD significantly impacts academic performance and emotional well‐being, increasing the risk of adverse outcomes in various domains (Livingston et al. 2018). Unsurprisingly, academic struggles often lead to frustration and feelings of inadequacy. CWD may find it challenging to keep up with their peers in traditional academic settings, leading to heightened anxiety and a negative self‐image. The constant challenges in educational and social settings can create intense feelings of sorrow and pain for CWD (Lavoie 2006). In the present context, the CWD were already clinically diagnosed with depression.

Finally, gender is included as a notable variable. DD is more frequently recognised in males than in females, and this disparity likely reflects a combination of biological, environmental, and referral‐related influences (Arnett et al. 2017). Examining gender distribution across functional profiles can therefore improve understanding of diagnostic patterns in the South African education system.

Overall, prior research converges on the outcomes that different types of DD may require different management approaches (Venkatesan 2017). A deeper understanding of how selected backgrounds and associated factors correlate with particular profiles could enhance clinical decision‐making, educator insight, and collaborative support approaches (Berninger and O'Malley May 2011; Geertsema et al. 2022).

### 1.1 Research Question

What are possible associations between background and etiological factors in subtypes of DD found in CWD in the South African classroom?

### 1.2 Research Objectives

The aim was to describe and compare etiological and background factors across the seven subtypes of DD in South African children. The study's objectives also sought to establish possible associations between these factors and the different subtypes.

## 2 Method

We employed a retrospective, cross‐sectional, and comparative research design using existing data from a South African dyslexia academy. The academy's principal was contacted to request access to the database for the study. All data in the database included parental consent and child assent for the anonymised datasets to be used for further research, training, and publication. Data were extracted from a background questionnaire with single questions and opportunities for follow‐up explanation, which were expanded upon during interviews. Ethical clearance was granted for the study.

Purposive sampling was used, and we included school‐aged participants with a standalone diagnosis of DD or co‐occurring disorders such as ADHD, ADD, and clinical depression (Stark et al. 2022). These co‐occurring disorders often represent the real‐life conditions in our classrooms. Participants met a set of criteria per the background information and interview reports to rule out exclusion factors, such as primary diagnoses of cognitive impairment, scholastic deprivation, and general reading and writing challenges, as described in the DSM‐5 and DSM‐5‐TR (American Psychiatric Association 2013). In essence, participants had to meet all four DSM‐5 criteria for an SLD diagnosis. The Dyslexia Assessment Test was administered to these participants across South Africa by HPCSA‐registered DD specialists (Stark et al. 2022).

### 2.1 Data Analyses

The sample size was 4370 school‐aged participants. The following total number of responses were included for each factor correlating with DD for the parent respondents who replied to the specifically related topic in regards to their child: late speech development (*n* = 4311), prior speech therapy received (*n* = 4062), audiological support sought (*n* = 4063), feelings of negative anger (*n* = 2772), feelings of negative frustration (*n* = 2934), negative emotional experiences (*n* = 3100), depression (*n* = 2599), and corresponding gender of their child (*n* = 4363). We captured the earmarked data sets in Excel sheets to determine, compare, and statistically equate the distinct factors. Each factor examined (e.g., audiological history, prior speech therapy, emotional and behavioural indicators, gender, and depression) was treated as an independently reported categorical variable derived directly from caregiver responses. No composite scores, indices, or latent constructs were created. Analyses focused on subtype‐specific differences in proportions for each factor independently, and associations or relationships between factors were not formally modelled.

The independent‐samples proportions test was used to test for statistically significant differences in proportions/percentages (*p* < 0.05). Where significant differences were identified, corresponding effect sizes were calculated and reported as Cohen's h for independent proportions, interpreted using conventional benchmarks (small ≈0.20; medium ≈0.50; large ≥ 0.80; Cohen 1988). Using G*Power software (Faul et al. 2007), the minimum required sample size for a small, medium, and large effect size to detect significant differences (two‐tailed tests) between the specific groups compared is 398 (199 in each group), 64 (32 in each group), and 28 (14 in each group), respectively, to obtain a statistical power of at least 0.8. However, many researchers have suggested that receiving the minimum sample size required to detect small effect sizes may be unnecessary, as finding a statistically significant result for a small effect may have statistical significance (*p* < 0.05), but not real‐world or practical significance (Baicus and Caraiola 2009; Peeters 2016). For example, a small effect size of 0.008 is flagged as statistically significant, and the question researchers raise is whether that would have any real‐world or practical significance. Thus, ignoring the minimum sample size requirements for small, medium, and large effect sizes, a minimum sample size of 64 is required for a medium effect size and 28 for a large effect size, as the aim was to obtain at least 64 responses.

Corrections for multiple comparisons, such as the Bonferroni adjustment, were not applied in this study. These corrections, while intended to reduce Type I error, are widely recognised as excessively conservative and can substantially inflate Type II error—thereby obscuring potentially meaningful patterns, especially in clinical and educational datasets where effect sizes may be subtle. Many researchers have explicitly argued against the routine use of Bonferroni‐type adjustments, noting that they make statistical conclusions dependent on the number of comparisons performed rather than on the strength of the evidence itself (Perneger 1998; Barnett et al. 2022). As Perneger (1998) emphasises, two researchers examining the same data could reach different conclusions solely because one performed more comparisons—a methodological inconsistency that undermines transparent interpretation. Given these concerns, and in line with contemporary recommendations against mechanical multiplicity corrections, we report exact *p*‐values and provide cautious, context‐based interpretation rather than applying rigid statistical penalties.

## 3 Results

### 3.1 Etiological and Background Factors in the Subtypes of DD

Results are presented in the order of the objectives of this study. An initial description of pre‐diagnostic parental behaviour and child characteristics regarding the specific factors and subsequent diagnoses is offered. This is followed by specific statistically significant comparisons between the subtypes to highlight and triangulate patterns for later discussion. (Although all subtypes were compared in this study, attention is only paid to significant comparisons for triangulation.) Of note, this manuscript elucidates the distinction among three concepts. Etiological factors pertain to causal mechanisms that play a role in DD (i.e., elements that are part of the causal pathway to onset). Determining etiological status requires evidence beyond associations found in cross‐sectional surveys (e.g., longitudinal, experimental, or genetic designs). Background factors denote pre‐existing contextual or historical traits of the child or family (e.g., demographics, family background, and early developmental environment) that help characterise the cohort and might influence risk or resource accessibility but are not considered causal according to our data. Associated factors denote variables that appear alongside DD in our survey findings (correlates/comorbidities). These relationships are explanatory and do not imply directionality or causation; they might reflect the effects of DD, common underlying factors, or confounding variables. Thus, our survey results are presented as background characteristics and related (correlational) factors, and we refrain from designating them as etiological unless backed by external causal proof.

#### 3.1.1 Audiological Visits Before Diagnosis

Of the total responses (*n* = 4063), 681 children visited an audiologist before their diagnosis. Of these, children with *dysphoneidesia* (visual‐and‐auditory DD subtype) represented the highest number (*n* = 285). Those with *dysnemkinphoneidesia* (motor‐visual–auditory DD) followed with *n* = 218. Visual or sight‐word/whole‐word decoding DD (*dyseidesia*) only provided four positive responses. Table 1 comprises the statistically significant subtype comparisons of audiological visit reports.

**TABLE 1:** Statistically significant DD subtype differences: Audiological visits before diagnoses.

Subtype comparison	Audiological visit before diagnosis (*n*/*N*, %)	*p*	Effect size
Dyseidesia vs. Dysnemkinphoneidesia	4/67 (6.0%) vs. 218/1159 (18.8%)	0.008	0.40
Dyseidesia vs. Dysnemkinphonesia	4/67 (6.0%) vs. 68/395 (17.2%)	0.019	0.36
Dyseidesia vs. Dysphoneidesia	4/67 (6.0%) vs. 285/1775 (16.1%)	0.026	0.32
Dyseidesia vs. Dysphonesia	4/67 (6.0%) vs. 91/559 (16.3%)	0.026	0.32

*Note:* Values are reported as *n*/*N* (%), where *N* represents the number of children within each subtype who provided a valid response to the audiological history item.

In all reported factorial comparisons, results are depicted and interpreted per this initial in‐depth elucidation of the subtype comparisons with the corresponding factor. For *dyseidesia* and *dysnemkinphoneidesia*, the *p*‐value 0.008 < 0.05 denoted that the discrepancy in the percentage of children diagnosed with *dyseidesia* (6.0%) who visited an audiologist before diagnosis yielded a statistically significant difference in comparison to those diagnosed with *dysnemkinphoneidesia* (18.8%). Effect sizes for the significant differences in Table 1 were small to moderate, suggesting consistent but modest subtype differences in pre‐diagnostic audiological visits.

#### 3.1.2 Late Speech Development

The second factor relates to parents' reports of delayed speech development in their CWD. Table 2 includes all the significant subtypes associated with this factor. Perusing the total number of completed responses to this sub‐section (*n* = 4311), 615 children were reported to exhibit late speech development according to the provided milestones. Of these, children with *dysphoneidesia* once again represented the highest number (*n* = 256; 41.6%). CWD with *dysnemkinphoneidesia* once more followed closely with *n* = 201 (32.7%). Motor‐based writing DD (*dysnemkinesia*) only provided a single yes‐answer (0.2%).

**TABLE 2:** DD significant subtype differences: Late speech development (*n* = 615; 14.1%).

Subtype comparison	Late speech development (*n*/*N*, %)	*p*	Effect size
Dysnemkinesia vs. Dysnemkinphoneidesia	1/52 (1.9%) vs. 201/1217 (16.5%)	0.005	0.56
Dysnemkinesia vs. Dysnemkinphonesia	1/52 (1.9%) vs. 71/418 (17.0%)	0.004	0.57
Dysnemkinesia vs. Dysphoneidesia	1/52 (1.9%) vs. 256/1875 (13.7%)	0.014	0.48
Dysnemkinesia vs. Dysphonesia	1/52 (1.9%) vs. 75/617 (12.2%)	0.026	0.44
Dysnemkinphoneidesia vs. Dysphoneidesia	201/1217 (16.5%) vs. 256/1875 (13.7%)	0.028	0.08
Dysnemkinphoneidesia vs. Dysphonesia	201/1217 (16.5%) vs. 75/617 (12.2%)	0.014	0.12

*Note:* Values are reported as *n*/*N* (%), where *N* represents the number of children within each subtype who provided a valid response to the late speech development item.

In Table 2, parent reports on their CWD diagnosed with *dysnemkineidesia* (1.9%) and *dysphonesia* (16.5%) differed significantly regarding late speech development. Comparably, *dysnemkinesia* (1.9%) and *dysnemkinphonesia* (17.0%) indicated a substantial difference, as the latter “auditory‐and‐motor” combined DD diagnosis is more strongly linked to later speech development. Another noteworthy link is highlighted by the substantive difference between *dysnemkinesia* (1.9%) and *dysphoneidesia* (13.7%), with similar significant differences between *dysnemkinesia* (1.9%) and *dysphonesia* (17.0%). For *dysnemkinphoneidesia* (16.5%) and *dysphoneidesia* (13.7%); *dysnemkinphoneidesia* (16.5%) and *dysphonesia* (12.2%); and *dysnemkinphonesia* (17.0%) and *dysphonesia* (12.2%), the *p*‐value also yielded significant differences regarding late speech development. Effect sizes in Table 2 ranged from small to medium, suggesting modest but occasionally more pronounced subtype differences in late speech development.

#### 3.1.3 Negative Anger

The third factor concerns indications of negative anger in the CWD. The overall number of positive responses to this question was 2772, of which *n* = 1002 (36.1%) children were reported to display feelings of anger. Of these, children with *dysphoneidesia*, yet again, represented the highest number (*n* = 424; 42.3%). CWD with *dysnemkinphoneidesia* followed with *n* = 317 (31.6%). Motor‐based written DD (*dysnemkinesia*) *was* least affected, with only 5 positive responses (0.5%). Negative anger associated with DD yielded no significant subtype differences in the proportion of participants diagnosed with the subtypes, and, accordingly, effect sizes are not reported. These outcomes differed slightly from the negative frustration factor.

#### 3.1.4 Negative Frustration

The fourth factor relates to parent/caregiver indications of negative frustration experienced by their CWD. Of the total number of responses in this section (*n* = 2934), 1477 CWD felt frustrated when dealing with specific subtypes of DD. Of these, children with *dysphoneidesia* represented the highest number (*n* = 639; 43.3%). Those with *dysnemkinphoneidesia* (motor‐visual–auditory DD) trailed with *n* = 465 (31.5%). *Dysnemkinesia* only had 10 positive responses (0.68%). Table 3 comprises all significant instances of children with negative frustration due to DD.

**TABLE 3:** DD significant subtype differences: Negative frustration (*n* = 2934; 67.14%).

Subtype comparison	Negative frustration (*n*/*N*, %)	*p*	Effect size
Dysnemkineidesia vs. Dysnemkinesia	23/38 (60.5%) vs. 10/28 (35.7%)	0.046	0.49
Dysnemkinphoneidesia vs. Dysphonesia	465/877 (53.0%) vs. 170/382 (44.5%)	0.005	0.19
Dysphoneidesia vs. Dysphonesia	639/1270 (50.3%) vs. 170/382 (44.5%)	0.046	0.12

*Note:* Values are reported as *n*/*N* (%), where *N* is the number of children within each subtype who provided a valid response to the negative‐frustration item.

Following Table 3, negative frustration associations with different types of DD are more evident than negative anger, albeit still not as prevalent as some of the other factors. *Dysnemkineidesia* (60.5%) and *dysnemkinesia* (35.7%) significantly declined from the combined visual‐motor DD to the motor‐based DD alone. Likewise, children with *dysnemkinphoneidesia* (53.0%) are considerably more frustrated than those with only *dypshonesia* (45%) based on the *p*‐value of 0.005 < 0.05 for this cohort. Moreover, regarding frustration on the side of those with diagnoses of *dysphoneidesia* (50.3%) and *dysphonesia* (45.0%), a statistically significant difference is also present. Effect sizes for the significant differences in Table 3 were predominantly small, with one comparison approaching a medium effect, suggesting generally modest subtype differences in levels of negative frustration.

#### 3.1.5 Depression

The fifth factor relates to indications of reported clinically diagnosed depression with a diagnosis of DD (Table 4). In the total number of responses in this section (*n* = 2599), CWD felt depressed when dealing with specific subtypes of DD. CWD with *dysphoneidesia* represented the highest number, *n* = 268 (50.76%). Those with *dysnemkinphoneidesia* trailed by *n* = 152 (28.79%). *Dysnemkinesia* had the fewest responses, being 0. Table 4 highlights all instances of the proportion of children with depression due to DD.

**TABLE 4:** DD significant subtype differences: Diagnosed depression associated with a diagnosis of DD (*n* = 2599; 59.47%).

Subtype comparison	Depression diagnosis (*n*/*N*, %)	*p*	Effect size
Dysnemkinesia vs. Dysnemkinphoneidesia	0/21 (0.0%) vs. 152/775 (19.6%)	0.024	0.94
Dysnemkinesia vs. Dysphoneidesia	0/21 (0.0%) vs. 268/1140 (23.5%)	0.011	1.02
Dysnemkinesia vs. Dysphonesia	0/21 (0.0%) vs. 62/335 (18.5%)	0.030	0.88
Dysnemkinphoneidesia vs. Dysnemkinphonesia	152/775 (19.6%) vs. 36/260 (13.8%)	0.037	0.17
Dysnemkinphoneidesia vs. Dysphoneidesia	152/775 (19.6%) vs. 268/1140 (23.5%)	0.043	0.10

*Note:* Values are reported as *n*/*N* (%), where *N* represents the number of children within each subtype who provided a valid response to the depression item. All depression diagnoses were clinically confirmed by qualified professionals.

Regarding the response “yes” for depression, there is a statistically significant difference in proportions between respondents experiencing depression with *dysnemkinesia* and those experiencing it with *dysnemkinphoneidesia* at the 0.05 significance level. Regarding *dysnemkinesia* and *dysnemkinphonesia*, as well as *dysphoneidesia* and *dysphonesia*, marginally statistically significant differences are present. Similarly, for *dysnemkinesia* and *dysphoneidesia, dysnemkinesia* and *dysphonesia, dysnemkinphoneidesia* and *dysnemkinphonesia, dysnemkinphoneidesia* and *dysphoneidesia, dysnemkinphonesia* and *dysphoneidesia* significant differences were determined. Effect sizes for the significant differences in Table 4 ranged from small to large, indicating that while some subtype differences in depression were modest, others reflected substantial variability in clinically diagnosed depression across DD subtypes.

#### 3.1.6 Prior Speech Therapy

The penultimate factor relates to indications of reported speech therapy prior to diagnoses of DD. The number of responses to this question was 4062; *n* = 1453 (35.8%) children visited an SLT before their diagnoses. Of these 1453 children with combined diagnoses, *dysphoneidesia* (*n* = 611; 42.1%) was the most common. CWD with *dysnemkinphoneidesia* followed with *n* = 430 (29.6%). *Dysnemkinesia* yielded only 16 speech therapy‐related answers (1.1%). Regarding this factor, only one significant difference between *dysnemkineidesia* (0.492) and *dyseidesia* (0.349) was calculated. This difference was associated with a small‐to‐moderate effect size (0.29), indicating a modest but meaningful difference in pre‐diagnostic speech‐language therapy exposure between these two subtypes.

#### 3.1.7 Gender

The final factor relates to gender differences in DD subtypes. Of the total number of 4363 parent responses who indicated their children's preferred gender, *n* = 1626 (37.3%) were identified as female, and *n* = 2737 (62.7%) as male. Table 5 presents the significant subtype comparisons for this factor, with the *p*‐values from the independent‐samples proportions test shown. Gender is reported as female; as gender was coded dichotomously, percentages for males can be inferred as the complement. Effect sizes reflect differences in the proportion of females across subtypes.

**TABLE 5:** DD significant subtype differences: Female (*n* = 1626; 37.3%).

Subtype comparison	Female (*n*/*N*, %)	*p*	Effect size
Dysnemkineidesia vs. Dysnemkinesia	35/63 (55.6%) vs. 19/52 (36.5%)	0.042	0.39
Dysnemkineidesia vs. Dysnemkinphoneidesia	35/63 (55.6%) vs. 411/1230 (33.4%)	< 0.001	0.47
Dysnemkineidesia vs. Dysnemkinphonesia	35/63 (55.6%) vs. 167/421 (39.7%)	0.017	0.33
Dysnemkineidesia vs. Dysphoneidesia	35/63 (55.6%) vs. 685/1904 (36.0%)	0.002	0.40
Dysnemkinphoneidesia vs. Dysnemkinphonesia	411/1230 (33.4%) vs. 167/421 (39.7%)	0.020	0.13
Dysnemkinphoneidesia vs. Dysphonesia	411/1230 (33.4%) vs. 279/621 (44.9%)	< 0.001	0.24
Dysphoneidesia vs. Dysphonesia	685/1904 (36.0%) vs. 279/621 (44.9%)	< 0.001	0.19

*Note:* Values are reported as *n*/*N* (%), where “female” reflects caregiver‐reported gender identification and *N* represents the number of children within each subtype with valid gender data. Reporting one category of a dichotomous variable avoids redundancy and unnecessary table density.

For *dyseidesia* and *dysnemkineidesia*, the proportions of females were 0.417 and 0.556, respectively. The difference in female representation between these two subtypes was not statistically significant (*p* = 0.107), indicating that the gender distribution of individuals diagnosed with *dyseidesia* did not differ significantly from that of individuals with *dysnemkineidesia*. Effect sizes for the significant differences in Table 5 ranged from small to moderate, indicating modest but consistent variation in the proportion of females across several DD subtype comparisons.

Of note regarding *dysnemkineidesia* and *dysnemkinesia*, the proportions of females were calculated as 0.556 for *dysnemkineidesia* and 0.365 for *dysnemkinesia*. With a *p*‐value < 0.05, the female distribution of individuals diagnosed with these two subtypes differs significantly. These significant numbers are also reflected for *dysnemkineidesia* and *dysnemkinphoneidesia*, *dysnemkineidesia* and *dysnemkinphonesia*, *dysnemkineidesia* and *dysphoneidesia*, *dysnemkinphoneidesia* and *dysnemkinphonesia*, *dysnemkinphoneidesia* and *dysphonesia*, and lastly *dysphoneidesia* and *dysphonesia*. The results indicate that gender distribution—operationalised as the proportion of females—varies significantly across subtypes of DD, which is unexpected given current research reports.

## 4 Discussion

This research study aimed to identify the background and etiological factors that differ significantly across the seven subtypes of DD, and to examine their specific impact on educators and SLTs, thereby facilitating a more precise understanding and management in the South African classroom. We selected audiological history, speech‐language development, emotional variables, and gender because each maps directly onto well‐established neurodevelopmental components that differentiate dyslexic profiles (auditory/phonological, visual‐orthographic, and motor/graphomotor systems). Auditory and temporal‐processing vulnerabilities—captured here by audiology visits and late speech—are tightly linked to phonologically based subtypes (dysphonesia and combined forms), with systematic reviews showing consistent auditory‐processing differences in reading‐impaired groups. Emotional difficulties (anger, frustration, and depression) are included because persistent reading failure and complex, multimodal subtype presentations predict elevated internalising symptoms and poorer school connectedness. Gender was retained, given documented sex‐related differences in neurodevelopmental presentation and diagnostic patterns. While socioeconomic status, language of instruction, and multilingual exposure are critical contextual moderators—especially in South Africa—they operate primarily at the environmental/educational level and were partially controlled for by the DSM‐5 exclusion of scholastic deprivation in the sample. Therefore, this study firstly isolates child‐level etiological indicators that plausibly distinguish subtype mechanisms and recommends that future multilevel work explicitly model socio‐economic and language factors as moderators.

The findings relating to the main objective of describing and comparing the different factors indicate that comparable background and etiological factors are illustrated across the multiple subtypes of DD, as reflected in Tables 1, 2, 3, 4, 5. Several context‐specific novel observations were made regarding the establishment of associations between the factors and the seven subtypes of DD. Regarding the *audiologist visits before diagnosis*, associations between *dyseidesia* (sight/whole word recognition) and the following subtypes were established: *dysnemkinphoneidesia*, *dysnemkinphonesia*, *dysphoneidesia*, and *dysphonesia*. This considerable difference suggests an association with children diagnosed with *dysnemkinphoneidesia* being more likely to have visited an audiologist before diagnosis than those diagnosed with *dyseidesia*. According to Werfel et al. (2020), CWD may present with deficits in speech perception in noise, which may explain some of these decoding deficit associations. For example, CWD may present with inadequate rapid temporal auditory information processing (Rahimi et al. 2022), defining the required audiological assistance as the primary type of dysphonesia (“auditory DD”) that is present in all these associations. A child with any audiological disorder is expected to present with delayed speech and language development (Dynak et al. 2021). Therefore, the following factor is *late speech development*.

For this factor, *dysnemkinesia* (the impaired written formation of graphemes) was found to significantly link to the following subtypes: *dysnemkinphoneidesia*, *dysnemkinphonesia*, *dysphoneidesia*, and *dysphonesia* (all related to the initial *dysnemkinesia* and to combined forms, including whole/sight‐word decoding and more complex auditory decoding). It was subsequently concluded that there are significant differences between *dysnemkinphoneidesia*, *dysphoneidesia*, and *dysphonesia* in relation to speech and language development. Late talkers unsurprisingly exhibit delays in these developmental areas. However, they may also present delayed literacy development in grammar, phonology, and reading accuracy (Dynak et al. 2021) – all of which may logically explain these findings. According to Kim (2021) CWD have less knowledge of vocabulary, syntax, and phonological language constructs. Moreover, for the subsequent factor of *prior speech therapy received*, the subtype *dysnemkineidesia, which* associates with *dysphonesia*, further ratifies the findings, as these challenges are part of the roles and practice scopes of SLTs (Wium and Louw 2013). Virlet et al. (2024) state that using a proprioceptive implicit instruction plan can improve CWD's linguistic capabilities (phonics, orthographic, and morphological instructions) by improving reading performance, eye movements, and the recognition of written words.

Following this devastating path of exacerbating factors as the CWD grows older, it is therefore not surprising that diagnosed *depression* is also locally significantly associated with a diagnosis of SLD, particularly between *dysnemkinesia* alone and the following three subtypes: *dysnemkinphoneidesia*, *dysphoneidesia*, and *dysphonesia*. In terms of associations, the statistically significant differences observed indicate that feelings of depression are present in all subtypes but are associated differently across specific subtypes of DD. These associations between the primary and combining subtypes of DD align with the findings of Sahoo et al. (2015) that reported approximately 30% of CWD experience comorbid depression, anxiety, and suicidal tendencies. Furthermore, Ihbour et al. (2021) concluded that CWD exhibit higher levels of anxiety, depression, and diminished self‐esteem compared to their non‐dyslexic peers. These findings underscore the profound psychosocial impact of DD and its subtypes on mental health. Moreover, the compounded subtypes (e.g., *dysnemkinphoneidesia* and *dysphoneidesia*) in our study also revealed more significant instances of depression due to a mixed involvement of auditory, visual, and written factors, reiterating Ihbour et al.'s (2021) findings that these factors are commonly reported alongside more severe challenges and feelings of unworthiness.

Regarding *negative anger*, there is no statistical significance among the various DD subtypes. However, even though no specific significant associations are present between different subtypes, it is essential to remember that CWD notably suffers from anger, frustration, and low self‐esteem (Kaisar 2020). This conclusion emphasises the importance of identifying these early to help them overcome their learning difficulties and optimally support them in class through empathetic understanding. Moreover, Wilmot et al. (2023) reported that many children with reading difficulties report experiencing heightened negative emotions such as anger, frustration, and sadness in the school context specifically. These negative feelings may intensify difficulties associated with DD, including academic performance and emotional well‐being (Livingston et al. 2018). Unsurprisingly, negative performance and emotions are closely interrelated to negative frustration.

*Negative frustration*—as reported in the present study—is significantly correlated with a diagnosis of DD, being particularly more present in children with *dysnemkinphoneidesia* (a multifaceted combination of phonetic and phonological awareness, motor, and sight word recognition challenges) than *dysphonesia* (involving mostly phonetic reading and spelling difficulties). Theodoridou et al. (2021) concluded in their study that school children with DD often face constant fear, anticipated failure, and frustration, adversely affecting their academic skills and self‐esteem, broadly correlating with our more specific findings. Prolonged exposure to these negative experiences can lead to increased frustration, lack of motivation, and, in some cases, aggressive behaviours. As such, these stressors may contribute to difficulties in coping with stress and frustration, combined with possible dysregulation, highlighting the link between negative frustration and the impact of DD on children's well‐being and academic achievement.

Additionally, our study reflects a statistical significance between the more complex *dysphoneidesia* and stand‐alone *dysphonesia* diagnosis as it relates to negative frustration in CWD. Authors Khan and Malik (2018) reiterate that most preschool CWD begin to develop emotional and behavioural problems as early reading instruction fails to match their learning style. This inability to meet the CWD's learning style and additional confusion about their expectations further creates frustration. These supportive findings make sense, as poor sight‐word recognition (*dyseidesia*) and the slightly more demanding levels of phonetic decoding (*dysphonesia*) begin to emerge in the early school phases, when phonological and phonemic awareness demands increase. Where both co‐occur (as in *dysphoneidesia*), frustration will increase.

Considering the gender distribution profiles and the significant proportions, as explained in terms of the *p*‐values, refer. Substantial differences are observed across the two genders for most DD subtypes. Taking into account more historical findings that substantiate the presence of gender differences regarding DD (Jiménez et al. 2011), it may be assumed that the generally agreed‐upon prevalence (3:1–4:1 ratios male: female) would justify the present results, concurring with Arnett et al. (2017) that males are more frequently diagnosed with DD compared to females. However, more recently, with the elucidation of SLD as a spectrum disorder, the numbers seem to be more equal than previously thought. The possibility of stealth DD and its less overt symptom presentation, for example, may account for underdiagnosis in the female groups and the overrepresentation of males (Arnett et al. 2017). The latter also presents different self‐accommodating strategies than their male peers (Liapi et al. 2024). Still, the overall results align with the more historical views. However, the unique diagnostic subtyping arising from the assessment tools may credibly contradict the traditional possible gender differences through the unique nature of the subtypes and the gender factors that may reveal these acceptable genetic and neurobiological discrepancies, psychosocial factors, and educational practices mentioned. Our findings, therefore, agree with Granocchio et al. (2023) in suggesting that, in general, males may be more susceptible to DD due to neurobiological, hormonal, and genetic factors. However, the finer subtyping of visual–, auditory–, and written DD should be further investigated in this regard.

## 5 Conclusion and Recommendations

This study has provided initial, novel evidence of significant associations between the described background, etiological factors, and the different subtypes of CWD in South Africa. By examining the intricate relationships between these factors and the subtypes, the study offers critical insights that can inform the development of targeted, individualised support and understanding within collaborative teams, such as educators and SLTs. The findings underscore the necessity of considering these underlying factors when recognising symptoms presenting in the classroom and considering empathetic management approaches. This may ensure that strategies are comprehensive and tailored to the unique needs of each subtype and the aggravating dynamics accompanying certain more complex combinations of DD subtypes. Collaboration with professionals to support these CWD regarding their emotional health is also highlighted for the collaborative team. Moreover, this research emphasises the importance of identifying and addressing the specific biological influences that may contribute to the manifestation of DD, ultimately supporting more precise and collaborative management.

Future research should aim to expand the participant pool to include more diverse populations and to consider additional background factors not included in this study (such as possible genetic links, specific language impairments, possible speech‐sound disorder associations, and specific classroom challenges).

## Funding

The authors have nothing to report.

## Disclosure

The authors declare that the principal of the dyslexia academy mentioned in this article is also the author of the validated assessment tool and a co‐author of this article. Some identifying changes have therefore been made to the academy and the tool.

## Ethics Statement

The Department of Speech‐Language Pathology and Audiology Ethics Committee, Faculty of Humanities, University of Pretoria, South Africa, granted ethical clearance (SLPA2024/05).

## Conflicts of Interest

The authors declare a conflicts of interest. The principal of the dyslexia academy mentioned in this article is also the author of the validated assessment tool, as well as a co‐author of this article. Some identifying changes have therefore been made to the academy, as well as the tool name.

## Data Availability

The data that support the findings of this study are available on request from the corresponding author. The data are not publicly available due to privacy or ethical restrictions. Data for the project has been uploaded to a data repository at the University of Pretoria for a minimum of 10 years. Access to this data may be granted by the corresponding author upon request.
